# Washout DNA copy number analysis by low-coverage whole genome sequencing for assessment of thyroid FNAs

**DOI:** 10.3389/fendo.2022.888072

**Published:** 2022-10-14

**Authors:** Linfeng Wu, Yuying Zhou, Yaoyao Guan, Rongyao Xiao, Jiaohao Cai, Weike Chen, Mengmeng Zheng, Kaiting Sun, Chao Chen, Guanli Huang, Xiaogang Zhang, Lijuan Zhai, Ziliang Qian, Shu-rong Shen

**Affiliations:** ^1^ Oncology and Hematology, Wenzhou Hospital of Integrated Traditional Chinese and Western Medicine, Wenzhou, China; ^2^ Department of Plastic Surgery, Sir Run-Run Shaw Hospital, Zhejiang University School of Medicine, Hangzhou, China; ^3^ Thyroid Surgery, The First Affiliated Hospital of Wenzhou Medical University, Wenzhou, China; ^4^ Hangzhou Catcher Bio Inc., Hangzhou, China; ^5^ Hongyuan Biotech Inc., Suzhou, China; ^6^ Prophet Genomics Inc., San Jose, CA, United States

**Keywords:** thyroid cancer, genome sequencing, washout-DNA, chromosome instability, LC-WGS

## Abstract

**Background:**

Papillary thyroid microcarcinoma (PTMC) is defined as a papillary carcinoma measuring ≤ 10 mm. The current management of PTMC has become more conservative; however, there are high-risk tumor features that can be revealed only postoperatively. For thyroid cancer, *BRAF* mutations and somatic copy number variation (CNV) are the most common genetic events. Molecular testing may contribute to clinical decision-making by molecular risk stratification, for example predicting lymph node (LN) metastasis. Here, we build a risk stratification model based on molecular profiling of thyroid fine needle aspiration (FNA) washout DNA (wDNA) for the differential diagnosis of thyroid nodules.

**Methods:**

Fifty-eight patients were recruited, FNA wDNA samples were analyzed using CNV profiling through low-coverage whole genome sequencing (LC-WGS) and *BRAF* mutation was analyzed using quantitative PCR. FNA pathology was reported as a Bethesda System for Reporting Thyroid Cytopathology (BSRTC) score. Ultrasound examination produced a Thyroid Imaging Reporting and Data System (TIRADS) score.

**Results:**

In total, 37 (63.8%) patients with a TIRADS score of 4A, 13 (22.4%) patients with a TIRADS score of 4B, and 8 (13.8%) patients with a TIRADS score of 4C were recruited after ultrasound examination. All patients underwent FNA with wDNA profiling. CNVs were identified in 17 (29.3%) patients. CNVs were frequent in patients with a BSRTC score of V or VI, including eight (47.1%) patients with a score of VI and five (29.4%) with a score of V, but not in patients with a score of III, II, or I (0%). *BRAF* mutation was not significantly correlated with BSRTC score. LN metastasis was found more frequently in CNV-positive (CNV+) than in CNV-negative (CNV–) patients (85.7% vs. 34.6%, odds ratio = 11.33, *p* = 0.002). In total, three molecular subtypes of thyroid nodules were identified in this study: 1) CNV+, 2) CNV– and *BRAF* positive (*BRAF*+), and 3) CNV– and *BRAF* negative (*BRAF*–). For the CNV+ subtype, 10 (83.3%) lesions with LN metastasis were found, including four (100%) small lesions (i.e. ≤ 5 mm). For the CNV– and *BRAF*+ nodules, LN metastases were detected in only seven (60.0%) larger tumors (i.e. > 5 mm). For CNV– and *BRAF*– tumors, LN metastasis was also frequently found in larger tumors only.

**Conclusions:**

It is feasible to identify high-risk LN metastasis thyroid cancer from FNA washout samples preoperatively using wDNA CNV profiling using LC-WGS.

## Introduction

Thyroid cancer is the most common malignant tumor in the endocrine system and in head and neck tumors (1). In recent years, the incidence of thyroid cancer has increased rapidly throughout the world, and the percentage of papillary thyroid microcarcinoma (PTMC) has presented the most growth and the fastest increase, but its mortality rate has remain stable (2). PTMC is defined by the WHO as papillary carcinoma of the thyroid measuring ≤ 10 mm (3). Most PTMCs are indolent and rarely develop into clinically significant thyroid cancer, and some are even asymptomatic for life; however, a small proportion of PTMCs are associated with highly aggressive features, and local invasion, lymph node (LN) metastasis, or distant metastases may occur at an early stage (4). Currently, treatment of PTMC can be controversial, focusing mainly on the necessity and scope of surgery. In accordance with guidelines issued by the American Thyroid Association in 2015, patients with low-risk PTMC are recommended to choose active surveillance (AS) rather than surgical treatment (5). The consensus statement on PTMC (2016) formulated by the Chinese Association of Thyroid Oncology (CATO) recommended that the need for surgical treatment of PTMC should be based on a preoperative risk assessment. For patients with low levels of psychological stress and no obvious risk factors (e.g. LN metastasis, high-risk molecular subtypes, family history, radiation exposure history, pathological high-risk subtypes), timely follow-up can replace surgery (6). However, the description of high-risk molecular subtypes in the statement remains unclear.

Fine needle aspiration (FNA) guided by ultrasonography has been used to obtain samples of thyroid neoplasms to prepare cytology smears for several decades (7, 8). FNA is the most accurate examination method for preoperative diagnosis of thyroid cancer and has been routinely used in clinical practice (9). However, by comparison with histopathology, about 10%–40% of thyroid cancer remains indeterminate using FNA (10). In the past 5–10 years, as the understanding of the molecular mechanisms underlying thyroid cancer has increased, several biomarkers have been developed for the diagnosis of thyroid cancer, including *RET*/*PTC*, *RAS*, and *BRAF* mutations (11). For example, since the early 2000s the *BRAF* gene mutation has been widely used as a hot diagnostic and prognostic marker for thyroid cancer in clinical practice, and the *BRAF* mutation test has been shown to improve diagnostic sensitivity to thyroid cancer (12). However, the *BRAF^V600E^* mutation occurs in only 50%–70% of papillary thyroid carcinomas (PTCs), and *BRAF* mutations can distinguish between only benign neoplasm and malignant thyroid tumors at present, but there is a lack of evidence supporting the correlation between the *BRAF* mutation and the malignancy of thyroid cancer (13). In addition, most of the studies involved in the current literature used postoperatively histological specimens for gene testing, which could not provide appropriate guidance for the surgical management of patients.

Chromosomal instability (CIN), which was first proposed by Boveri nearly a century ago, refers to changes in chromosome number and structural aberrations caused by errors in chromosome separation in tumor cells during mitosis and was considered a hallmark of cancer (14). CIN is prevalent in various tumor types and is a manifestation of heterogeneity within tumors. In addition, CIN has been associated with metastasis, drug resistance, and poor prognosis in a wide range of cancers (15). Studies have shown that CIN is also common in thyroid cancer, including chromosomal rearrangement, copy number variations, and focal amplifications. Recent studies have shown that different types of thyroid cancer have chromosome copy number abnormalities, manifested as loss of heterozygosity (LOH), and chromosome 9p, 13p, and 22q LOH frequently occurs in thyroid tumor tissues, accounting for approximately 40% of cases (16). Patients with poorly differentiated thyroid cancer mostly have chromosomal abnormalities and loss of chromosome 9 copy number, and the 2-year survival rate is< 30% (17). In recent years, next-generation sequencing and analysis methods have greatly promoted the identification and cataloging of somatic cell copy number variations (CNVs), providing new possibilities to better detect dynamic changes of CIN (18). Scheinin et al. developed a low-coverage whole genome sequencing (LC-WGS) assay, which is a simple, economical, and reliable CNV identification technique (19).

The present study investigated the incidence of CIN in FNA washout samples from thyroid nodules. In addition, we investigated the significance of CIN in washout samples for the diagnosis of thyroid carcinoma.

## Methods

### Patient characteristics and ethics statement

A cohort study was conducted involving 58 patients with thyroid nodules (**Table 1**), with the approval of an ethics committee (approval number 2021-K006) and the informed consent of all patients. Informed consent was obtained from every patient prior to clinical trial participation. All patients underwent fine needle aspiration biopsy (FNAB) sampling at admission to identify aberrations and cytology of the chromosomes.

**Table 1:** Patient characteristics.

Parameter	Subtype	Frequency	Percentage
Gender	Male	11	19.0
	Female	47	81.0
Age (years)	≤ 45	28	48.3
	> 45	30	51.7
CNV	Negative	41	70.7
	Positive	17	29.3
*BRAFV600E*_washout	Negative	26	44.8
	Positive	32	55.2
*BRAFV600E*_tissue	Negative	24	41.4
	Positive	34	58.6
Clinical Pathology	Benign	15	25.9
	PTC	40	69.0
	Udx	3	5.2
Lymph node	Negative	19	47.5
	Metastasis	21	52.5
TIRADS	4a	37	63.8
	4b	13	22.4
	4c	8	13.8
Thyroid nodule size (mm) (ultrasound)	< 5	5	8.6
	5–10	31	53.4
	> 10	22	37.9
BSRTC	VI	15	25.9
	V	19	32.8
	III	6	10.3
	II/I	18	31.0
Multi_foci	1	29	72.5
	> 1	11	27.5
Tumor size (mm)	≤ 5	14	35.0
	> 5	26	65.0
ETE	Negative	37	92.5
	Positive	3	7.5

CNV, copy number variation; PTC, papillary thyroid cancer; Udx, undiagnostic; TIRADS, Thyroid Imaging Reporting and Data System; BSRTC, Bethesda System for Reporting Thyroid Cytopathology; ETE, extrathyroidal extension.

### DNA extraction

The washout samples were centrifuged at a g-force of 300g for 10 min, and the washout DNA (wDNA) was isolated using a QIAGEN (Hilden, Germany) circulating nucleic acid kit.

### Low-coverage whole genome sequencing

LC-WGS was carried out as previously described (20). Libraries were prepared using the KAPA HyperPrep Kit (Roche, Basil, Switzerland) with custom adapters [Integrated DNA Technologies (IDT) and Broad Institute], starting with 3–20 ng of cell-free DNA input (median 5 ng), or approximately 1,000–7,000 haploid genome equivalents, for low-pass whole genome sequencing. Up to 22 libraries were pooled and sequenced using 150-bp pair-end runs over 1 × lane on a HiSeq X Ten (Illumina Inc., San Diego, CA, USA). Segment copy numbers were derived using a customized workflow ultrasensitive chromosomal aneuploidy detector (UCAD). The sample was excluded if the median absolute deviation of copy ratios (log_2_ ratio) between adjacent bins, genome-wide, was 0.38, suggesting poor-quality sequence data.

### BRAF^V600E^ amplification refractory mutation system assay

Analysis of the *BRAF^V600E^* mutation was performed using an amplification refractory mutation system, that is, using a BRAF Gene V600E Diagnostic kit (AmoyDx, Xiamen, China) on a real-time PCR system in accordance with the manufacturer’s instructions.

### Statistical analyses and data visualization

The wDNA was isolated and sequenced using a HiSeq X Ten. At least 10 paired-end reads were collected per sample. The sequences were then aligned to the human reference genome hg19. The genomic coverage and depth were calculated using the Samtools software package (20). The average coverage was calculated for each 200k bin. Data were normalized by *z*-score transformation using the following formula:


(Formula 1) $${\text{Coverage}}_{\text{normalized}}=\frac{{\text{coverage}}_{\text{raw}}\;-\text{ mean}\left({\text{coverage}}_{\text{controls},\text{ raw}}\right)}{\text{stdev}\left({\text{coverage}}_{\text{controls},\text{ raw}}\right)}$$

Significant genomic breakpoints and CNVs were identified using the circula binary segmentation algorithm in the R package “DNACopy” (21). A *p*-value of< 0.05 was considered statistically significant. The chi-squared test was used to analyze categorical variables. All statistical analyses were performed by SPSS software (version 17.0). The proportional trend test was used to analyze the correlation between positive UCAD screening and clinicopathological parameters. Data were presented as mean and standard deviations, median and quartile ranges, and hazard ratios or odds ratios (ORs) with 95% CIs. Missing data were deleted from the analysis. All analyses were performed using R software, version 3.4.3. The anonymized data and the R code used for statistical analysis can be provided on request.

## Results

### Patient characteristics

**Figure 1:**
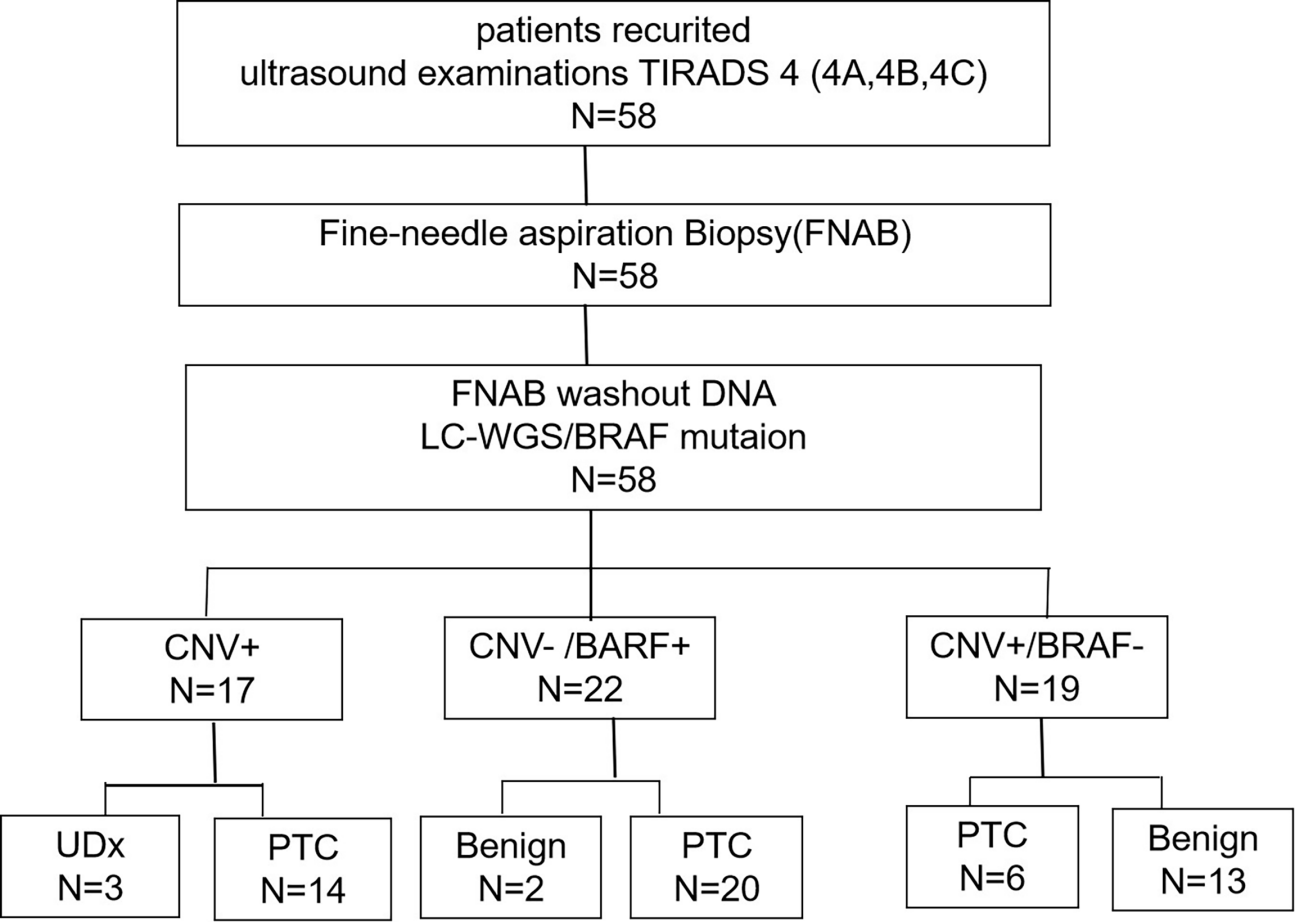
The STARD flowchart for participant recruitment.

At the time of manuscript preparation, 58 patients with potentially malignant tumors according to an ultrasound examination Thyroid Imaging Reporting and Data System (TIRADS) score of 4 (i.e. 4A, 4B, 4C), and who had a clinician’s recommendation for and whom had consented to FNAB, were recruited. Among the 58 patients, 47 (81.0%) were women and 11 (19.0%) were men; 37 (63.8%) had a TIRADS score of 4A, 13 (22.4%) a TIRADS score of 4B, and eight (13.8%) a TIRADS score of 4C. The numbers of patients with thyroid nodule sizes measuring < 5 mm, 5 mm–10 mm, and > 10 mm were five (8.6%), 31 (53.4%), and 22 (37.9%), respectively. The specific malignant indications of recruited patients evaluated by ultrasound as shown in **sTable 1**, including hypoechogenicity, irregular margins, calcifications and taller-than-wider shape. Furthermore, 11 (19.0%) patients with multiple lesions were identified. The tumor size was also measured in patients who underwent surgery. The STARD (Standards for the Reporting of Diagnostic Accuracy Studies) flow diagram is shown in **Figure 1**.

### Cytology reports of fine needle aspirations

All patients were further investigated using fine needle investigations. Fifteen (25.9%) patients were reported as having a Bethesda System for Reporting Thyroid Cytopathology (BSRTC) score of VI, 19 (32.8%) patients with a BSRTC score of V, and six (10.3%) patients with a BSRTC score of III. The remaining 18 (31.0%) patients were reported as having a BSRTC score of I/II (**Table 1**).

### Pathological reports

As shown in **Table 2**, 40 patients underwent surgical treatment, including all 15 (100%) patients who had a BSRTC score of VI, 17 out of the 19 (89.5%) patients who had a BSRTC score of V, and three out of the six (50%) patients who had a BSRTC score of III (**Table 2**). All patients were confirmed with malignancy after pathological examinations. Five out of the 18 (27.8%) patients who had a BSRTC score of I/II also underwent surgical treatment. In addition, three out of 40 (7.5%) patients were confirmed with extrathyroidal extension (ETE): two patients with a BSRTC score of VI and one patient with a BSRTC score of III.

**Table 2:** Pathology findings for different BSRTC score patient groups.

		BSRTC, *n* (%)				Total, *n* (%)
		VI	V	III	II/I	
Clinical pathology	PTC	15 (100)	17 (89.5)	3 (50)	5 (27.8)	40 (69.0)
No Surgery	0 (0)	2 (10.5)	3 (50)	13 (72.2)	18 (31.0)

### Washout cells showed high-consistency BRAF*^V600E^* mutations status with matched tissue

As shown in **Table 3**, of the 54 samples with wDNA and tissue DNA matching available, high levels of consistency of *BRAF* mutations were found. The FNA washout predicted 31 out of 34 (91.2%) *BRAF* mutations. Furthermore, wDNA revealed one additional *BRAF* mutation. The overall consistency between washout and matched tissue was 93.1%.

**Table 3:** Comparison between washout and matched tumor tissues.

		*BRAF^V600E^* _washout		Percentage
		Mutated	Wild type	
*BRAF^V600E^* _tissue	Mutated	31	3	91.2
	Wild type	1	23	95.8
Overall percentage				93.1

### Washout cells showed copy number variations

Chromosomal aberrations were frequently identified on chromosomes 22 and 17. Chr22q deletions were present in 11 (19.0%) samples. Chromosome 17 gains were identified in three (5.2%) samples. In patients who underwent surgery, all patients with deletion of chr22q were diagnosed with PTC. All chromosome 17 gains were among patients with a BSRTC score of II. In patients with chromosome 17 gains, gains continued to be observed during the follow-up period. No pathological examinations were reported.

**Figure 2:**
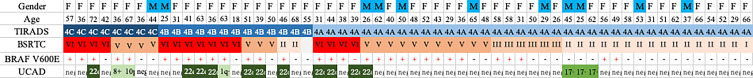
Overview of genetics changes and patient characterizations.

Frequent CNVs were found in patients with a higher BSRTC score, including eight (53.3%) patients with a score of VI and five (26.3%) patients with a score of V. Fewer chromosomal copy number changes were identified in patients with a score of III (0/6 = 0%) or I/II (3/18 = 16.7%). The results indicate that chromosomal instabilities may be associated with tumor aggressiveness (**Figure 2**).

### The adding of washout copy number variations profiling increased detection sensitivity

As shown in **Table 4**, *BRAF^V600E^* mutations were found in 55.2% and 58.0% of the samples in our dataset and The Cancer Genome Atlas (TCGA) dataset, respectively. Somatic copy number alterations (SCNAs) were found in 29.3% and 16.1% samples in our dataset and of the TCGA PTC dataset, respectively. The addition of SCNA increases the detection positive rate to 62.1% and 65.1% for our dataset and the TGCA PTC dataset, respectively.

**Table 4:** The addition of washout copy number profiling increases cancer detection sensitivity.

Group	Event	Frequency (%)
This study	*BRAF^V600E^* or SCNA	62.1
	*BRAF^V600E^*	55.2
	Washout SCNA	29.3
TCGA	*BRAF^V600E^* or SCNA	65.1
	*BRAF^V600E^*	58.0
	Washout SCNA	16.1

### Variations in copy number and tumor size correlated with lymph node metastasis in papillary thyroid carcinoma

It is difficult to determine preoperative LN metastasis of PTC in clinical practice. As shown in **Table 5**, we found that the CNV was statistically correlated with LN metastasis [*p* = 0.002, OR = 11.33 (95% CI 2.07 to 62.11]). The tumor size was also correlated with LN metastasis [*p* = 0.026, OR = 4.72 (95% CI 1.15 to 19.41]). However, there was no significant correlation between the *BRAF* mutation, ETE, and LN metastasis [*p* = 0.689, OR = 1.6 (95% CI 0.31 to 8.30) and *p* = 0.614, OR = 1.90 (95% CI 0.16 to 22.75), respectively].

**Table 5:** The correlation between CNV, *BRAF* and tumor size with LN metastasis of PTC.

		N stage, *n* (%)		Total (*n*)	OR (95% CI)	*p-*value
		N0	N1			
CNV	CNV–	17	9 (34.6)	26	11.33 (2.07 to 62.11)	0.002
	CNV+	2	12 (85.7)	14		
*BRAF* (mutation)	*BRAF*–	4	3 (42.9)	7	1.6 (0.31 to 8.30)	0.689
	*BRAF*+	15	18 (54.8)	33		
Size (mm)	≤ 5	10	4 (28.6)	14	4.72 (1.15 to 19.41)	0.026
	> 5	9	17 (65.)	26		
ETE	Negative	18	19 (51.4)	37	1.90 (0.16 to 22.75)	0.614
	Positive	1	2 (66.7)	3		

CNV, copy number variation; ETE, extrathyroidal extension.

**Figure 3:**
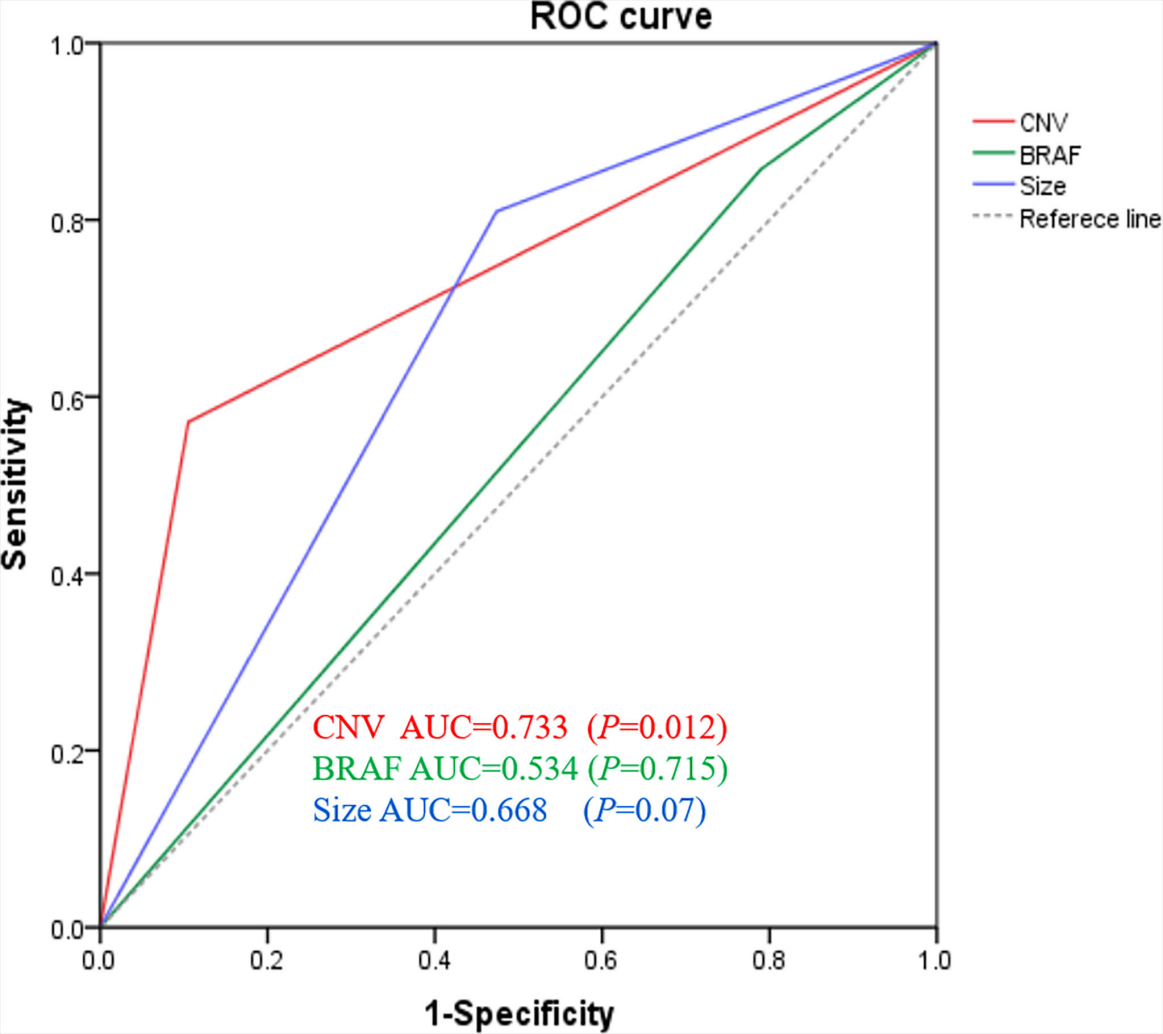
Genomic classifier to predict lymph node metastasis risk. ROC of CNV, *BRAF*, and tumor size of lymph node prediction in all surgical patients (*n* = 40). ROC, receiver operating characteristic; AUC, area under curve.

The receiver operating characteristic curves were plotted to identify the diagnostic value of the three variables to predict LN metastases. As shown in **Figure 3**, CNV gave the best performance for the prediction of LN metastasis. The order of these three variables to predict LN metastasis was CNV followed by tumor size followed by *BRAF*. The area under the curve (AUC) of CNV was 0.733 (95% CI 0.574 to 0.892), which was higher than that of tumor size (AUC 0.668, 95% CI 0.496 to 0.840) and *BRAF* (AUC 0.534, 95% CI 0.352 to 0.715) for all patients In addition, as shown in **Table 6**, the prediction performance is significantly higher than that of BRAF (p=0.015).

**Table 6:** CNV, *BRAF*, and tumor size in predicting lymph node metastasis.

	AUC (95% CI)	TN	TP	FN	FP	PPV (%)	NPV (%)	Sensitivity (%)	Specificity (%)	Accuracy (%)
CNV	0.733 (0.574 to 0.892)	17	12	9	2	85.7	65.3	57.1	89.5	72.5
*BRAF*	0.534 (0.352 to 0.715)	4	18	3	15	54.5	57.1	85.7	21.1	55
Size	0.668 (0.496 to 0.840)	10	17	4	9	65.4	71.4	81	52.6	67.5

AUC, area under curve; TN, true negative; TP, true positive; FN, false negative; FP, false positive; PPV, positive prediction value; NPV, negative prediction value. CNV vs. BRAF, p=0.015.

A binary logistic regression model was established to evaluate the effects of CNV, *BRAF* mutation, and tumor size on LN metastasis in patients with PTC. As shown in **Table 7**, the final logistic model was statistically significant. The model could correctly classify 72.5% of the subjects. Sensitivity and specificity were 57.1% and 89.5%, respectively. The positive predictive value and negative predictive value were 85.7% and 65.4%, respectively. Among the three independent variables included in the model, CNV and tumor size were statistically significant, and their ORs were 14.122 (95% CI 2.099 to 95.35) and 6.624 (95% CI 1.088 to 36.073), respectively.

**Table 7:** Binary logistic regression model for LN metastasis in PTC.

				N stage predicted		Percentage correct
				N0	N1	
N stage observed			N0	17	2	89.5
N1	9	12	57.1
Overall percentage						72.5
Variables in the equation.						
	B	SE	Wald	df	Sig.	OR (95% CI)
CNV	2.648	0.973	7.41	1	0.006	14.122 (2.099 to 95.035)
Size	1.835	0.893	4.22	1	0.040	6.264 (1.088 to 36.073)
Constant	–1.908	0.825	5.345	1	0.021	0.148

Binary logistic regression model for LN metastasis.

B, regression coefficient beta; SE, standard error; Wald, Wald chi-square value; df, degree of freedom; Sig., significance, p value.

### Variations in copy number correlated with lymph node metastasis in papillary thyroid microcarcinoma

We further classified PTC into three molecular subtypes based on the mutation of CNV and *BRAF*: 1) CNV positive (CNV+), 2) CNV negative (CNV–) and *BRAF* positive (*BRAF*+), and 3) CNV– and *BRAF* negative (*BRAF*–).

As shown in **Table 8**, for PTMC with a tumor size< 5 mm, LN metastasis was identified in four (100%) CNV+ patients but was not identified in CNV– patients (OR > 10, *p* = 0.001). For PTMC with a tumor size > 5 mm, LN metastasis was found in eight (80.0%) CNV+ patients and in seven (63.6%) CNV– and *BRAF*+ patients (OR 2.283, *p* = 0.635).

In summary, copy number variations and a tumor size > 5 mm could predict high-risk PTC with LN metastasis risk, whereas *BRAF* mutation showed no prediction value for LN metastases.

## Discussion

Thyroid nodules have become a common clinical problem, and differentiated thyroid cancer is becoming more and more prevalent (5). The differential diagnosis of thyroid nodules and the risk stratification of malignancy require multidisciplinary expertise, including local ultrasound practice and specific FNA cytology (22). Even in this multidisciplinary setting, some of the cytopathological results of FNA are still undetermined as benign or malignant (23). However, there is growing evidence that this limitation can be overcome by using molecular diagnostic methods through comprehensive genomic analysis (11). There is, however, no suitable biomarker to indicate the malignant degree of thyroid cancer and so it is of great clinical significance to find biomarkers with high specificity and sensitivity to establish a rapid, economic and reliable detection technology.

Molecular diagnosis of FNA is an important complement to FNA cytology; it can significantly reduce unnecessary surgery and help to better determine whether or not surgery is required for thyroid nodules in patients with uncertain FNA cytology. For example, the greatest benefit has come from the use of *BRAF* mutations in the diagnosis of malignancies, as *BRAF* mutations are highly specific for malignancies when detected using well-validated techniques (24). However, more than 10% of thyroid cancers are wild-type *BRAF*, and these tumors may be more aggressive, as they have been reported to harbor chromosomal aberrations (25).

In this study, 58 thyroid FNA biopsy samples were analyzed using LC-WGS, and chromosomal changes and *BRAF^V600E^* mutations were identified. We found a coincidence rate of 93.1% between *BRAF* mutations detected in wDNA using this technique and the results of routine clinical tests. Furthermore, we found that an additional three (5.1%) patients were identified as wild-type *BRAF* in routine tests, but *BRAF^V600E^* mutations were identified using LC-WGS profiling. Combination genetic testing can improve the detection rate of *BRAF* mutations.

The treatment of low-risk PMTC (T1aN0M0) has become a major clinical problem in recent years. Although many AS clinical trials for low-risk PTMC have reported favorable outcomes, not all PTMCs are suitable for AS (26). According to the current AS guidelines for PTMC, indications include the presence of LN metastases or distant metastases, aggressive subtypes, and suspected invasion of important structures in the neck (recurrent laryngeal nerve or trachea). Tumors located near these structures should be treated immediately with surgery. Among these tumors, cervical LN metastasis is a risk factor for an increased rate of recurrence in patients with PTMC (27). There are many factors for central LN metastasis of PTMC, including age, tumor diameter, and thyroid capsule invasion (28). Potential central LN metastases have been found to occur in the early stage of PTMC, especially in the central region (29). Central LN dissection may increase the risk of recurrent laryngeal nerve and parathyroid gland injury, and these complications are often the main factors in medical disputes (30, 31). Furthermore, this discomfort can have a huge impact on the patient’s subsequent quality of life and mental health. Hence, a correct preoperative evaluation is important in deciding whether or not to perform prophylactic central LN dissection (32). So far, there is no effective biomarker for the preoperative evaluation of LN metastases.

In this study, we determined that CNV was significantly associated with LN metastasis in PTC, but *BRAF* was not, especially for PTMC ≤ 5 mm. As shown in **Table 8**, LN metastases were confirmed after surgery in all four patients with positive CNV. Among these patients, a patient with a TIRADS score of 4B and a BSRTC score of II who did not receive timely surgical intervention was, on subsequent surgery, confirmed to have PTMC with LN metastasis. Among CNV– patients, none underwent surgery or developed LN metastasis. In addition, we followed up two patients who were CNV– and *BRAF*+ without surgery for 14 and 18 months, respectively, and found no abnormalities. These findings suggest that patients with PTMC< 5 mm are at a higher risk of LN metastasis if CNV is positive, whereas CNV– patients belong to a low-risk subtype and AS can be used instead of surgery.

**Table 8:** Association of three molecular subtypes with LN metastasis of PTC.

LN size	CNV+	CNV– and *BRAF*+	CNV– and *BRAF*–	OR (95% CI)	*p*-value
≤ 5 mm					
N0	0	9	1	a	0.001
N1	4	0	0	(CNV+ vs. CNV– and *BRAF*+)	0.001
> 5 mm					
N0	2	4	3	2.283 (0.316 to 16.393)	0.366
N1	8	7	2	(CNV+ vs. CNV– and *BRAF*+)	0.635
All sizes					
N0	2	13	4	11.1 (1.92 to 66.67)	0.006
N1	12	7	2	(CNV+ vs. CNV– and *BRAF*+)	0.003

Furthermore, in this study, tumor size was found to be a risk factor for LN metastasis in PTC (OR = 4.72). For PTMC ≤ 5 mm, the incidence of LN metastasis was 28.6%. In the absence of CNV+, there is no evidence of LN metastasis. However, in our study, when the tumor size was > 5 mm, the proportion of LN metastasis increased significantly (65.3%). LN metastasis tended to occur independently of CNV (**Table 5**). These results were consistent with previous reports on the relationship between tumor size and LN metastasis. For example, in a retrospective cohort study, central LN metastases were found to be associated with tumor size (> 5 mm) but not with the *BRAF* mutation (33).

Genomic instability, generally considered to be a promoter of solid tumor progression, usually occurs in three forms: microsatellite instability, aneuploidy, or intrachromosome instability (34). Although much is known about the underlying mechanisms of microsatellite instability and aneuploidy, less is known about the molecular basis of intrachromosome instability (35). In fact, it now appears that intrachromosomal instability can be subdivided into several independent forms, each of which may have its own molecular origin, which is evident when different analytical methods are used (36). In this study, we clarified features of genomic instabilities described in thyroid carcinomas. As shown in **Figure 2**, 14 out of 58 (24.1%) cases presented chromosome instability detected by LC-WGS, including chromosome 22q loss, chromosome 17p loss, and chromosome 1q gain. Most patients with positive CIN underwent surgery and were confirmed as having thyroid cancer by postoperative pathology.

Frequent allelic deletions of, for example, chromosome 22q could suggest genetic areas to be evaluated to detect the presence of suppressor genes associated with follicular thyroid cancer (37). The consistency of the Ch22q deletion pattern in PTC suggests that this genetic lesion may represent a distinct subgroup of these tumors. The consistency of the chromosome 22q deletion pattern in PTC suggests that the genetic lesion may represent a unique subtype of these tumors.

In this context, it should be noted that a large number of Chr22q deletions are associated with tumor aggressiveness and a distinct subtype of malignant follicular carcinoma and, therefore, may indicate a precursor lesion. However, we were unable to associate any clinical or pathological parameters with specific CNV populations, other than the statistically significant association between chromosome 22q deletion groups and young age. It is worth noting that these two wild-type *BRAF* PTCs support the notion that PTCs can broadly belong to follicular or papillary neoplasms, each with different molecular and clinical characteristics.

Taken together, in the study, LC-WGS detected CNV and *BRAF* mutation status of FNA wDNA. We found that the detection of the CNV and *BRAF* mutation increased the sensitivity of FNA. In addition, CNV has a significant correlation with LN metastasis of PTC, especially for PTMC< 5 mm, which is of clinical significance. We also found that tumor size was a risk factor for LN metastasis. Therefore, we conclude that CNV and a larger tumor (i.e. > 5 mm) are risk factors for PTC LN metastases. CNV detection can be used for the preoperative risk stratification of PTC.

## Data availability statement

The datasets presented in this study can be found in online repositories. The names of the repository/repositories and accession number(s) can be found in the article/**Supplementary Material**. The raw sequence and processed data fles are available through the National Omics Data Encyclopedia database (https://www.biosino.org/node/search) with accession number OEP003680.

## Ethics statement

The studies involving human participants were reviewed and approved by the Institutional Ethics Committee of Wenzhou Hospital of Integrated Traditional Chinese and West Medicine of Zhejiang Province. The patients/participants provided their written informed consent to participate in this study. Written informed consent was obtained from the individual(s), and minor(s)’ legal guardian/next of kin, for the publication of any potentially identifiable images or data included in this article.

## Author contributions

LW, YZ, YG, RX, JC, WC, MZ, KS, CC, GH, XZ, and LZ participated in the design of the study and performed the statistical analysis; LW, YZ, ZQ, and S-rS drafted the manuscript. All authors read and approved the final manuscript.

## Funding

Major science and technology projects of Wenzhou science and Technology Bureau grant number: ZY2021008.

## Conflict of interest

Authors LZ and XZ were employed by Suzhou Hongyuan Biotech Inc. and Hangzhou Catcher Bio Inc. Author ZQ was employed by Suzhou Hongyuan Biotech Inc. and Prophet Genomics Inc.

The remaining authors declare that the research was conducted in the absence of any commercial or financial relationships that could be construed as a potential conflict of interest.

## Publisher’s note

All claims expressed in this article are solely those of the authors and do not necessarily represent those of their affiliated organizations, or those of the publisher, the editors and the reviewers. Any product that may be evaluated in this article, or claim that may be made by its manufacturer, is not guaranteed or endorsed by the publisher.
